# The safety and anti-tumor effect of multiple peptides-pulsed dendritic cells combined with induced specific cytotoxic T lymphocytes for patients with solid tumors

**DOI:** 10.3389/fimmu.2023.1284334

**Published:** 2023-10-24

**Authors:** Xuan Zhao, Zhen Zhang, Chunli Wen, Jianmin Huang, Shuangning Yang, Jinyan Liu, Huizhen Geng, Bing Peng, Zibo Li, Yi Zhang

**Affiliations:** ^1^ Biotherapy Center & Cancer Center, The First Affiliated Hospital of Zhengzhou University, Zhengzhou, Henan, China; ^2^ State Key Laboratory of Esophageal Cancer Prevention & Treatment, Zhengzhou University, Zhengzhou, Henan, China; ^3^ Henan Key Laboratory for Tumor Immunology and Biotherapy, Zhengzhou, Henan, China; ^4^ Hebei Bio-High Technology Development Co., LTD. Shijiazhuang, Hebei, China; ^5^ School of Life Sciences, Zhengzhou University, Zhengzhou, Henan, China

**Keywords:** cytotoxic T lymphocytes, peptide, dendritic cell, efficacy, safety

## Abstract

**Objective:**

The aim of this study was to explore the safety and efficacy of multiple peptide-pulsed autologous dendritic cells (DCs) combined with cytotoxic T lymphocytes (CTLs) in patients with cancer.

**Methods:**

Five patients diagnosed with cancer between November 2020 and June 2021 were enrolled and received DC-CTLs therapy. Peripheral blood was collected and antigenic peptides were analyzed. The phenotype and function of DC-CTLs and the immune status of patients were detected using flow cytometry or IFN-γ ELISPOT analysis.

**Results:**

DCs acquired a mature phenotype and expressed high levels of CD80, CD86, CD83, and HLA-DR after co-culture with peptides, and the DC-CTLs also exhibited high levels of IFN-γ. Peripheral blood mononuclear cells from post-treatment patients showed a stronger immune response to peptides than those prior to treatment. Importantly, four of five patients maintained a favorable immune status, of which one patient’s disease-free survival lasted up to 28.2 months. No severe treatment-related adverse events were observed.

**Conclusion:**

Our results show that multiple peptide-pulsed DCs combined with CTLs therapy has manageable safety and promising efficacy for cancer patients, which might provide a precise immunotherapeutic strategy for cancer.

## Introduction

Cancer is a major disease that threatens human health and quality of life (1). Despite encouraging results from traditional treatment methods, their efficacy is moderate and the recurrence rate remains high. Moreover, serious side effects induced by treatments such as chemotherapy and radiotherapy make it less likely for patients to receive a long-term treatment. Therefore, there is an urgent need for effective, low-toxicity, and more tolerable treatment methods (2–4). Adoptive cell therapy, a promising therapeutic modality that stimulates or enhances patients’ immune function, has unique advantages in reducing the tumor treatment-related side effects, preventing recurrence and metastasis, improving the quality of life, and extending the curative effect (5, 6). Adoptive cell therapy can be mainly divided into two types in clinical applications: non-specific cell therapy, such as cytokine-induced killer (CIK) cell therapy and natural killer (NK) cell therapy, and specific cell therapy, such as T cell receptor (TCR) gene-transduced T cell therapy and chimeric antigen receptor (CAR)-T cell therapy (7–10). Specific anti-tumor immune therapies show promising clinical efficacy in some clinical studies. CAR-T cell therapies have been approved treating B cell malignancies (11, 12). Among these antigen-specific adoptive T cell therapies, dendritic cells (DCs) plus cytotoxic T lymphocytes (CTLs) therapy has unique advantages, and can effectively strengthen the immune function by activating the active and passive immune mechanism and improving the immune status of patients to achieve an equilibrium condition (13, 14).

DC-CTLs therapy includes the infusion of DCs loaded with tumor antigens and CTLs generated by coculturing T cells with tumor antigen-pulsed DCs. DCs are central to the initiation, regulation, and maintenance of immune responses. DCs containing antigens can trigger antigen specific T cells to produce immune responses both *in vitro* and *in vivo* (15, 16). After co-incubation with DCs, T cells kill antigen-expressing cells. These CTL-producing cytokines can kill tumor cells directly when infused into patients and exhibit high proliferative ability and cytolytic activity *in vitro* and *in vivo* (17). At present, most of tumor-specific T cell therapies, including TCR-T cells, CAR-T cells, and DC-CTLs, focusing on a single target, and their clinical application has certain limitations. In this study, we designed multiple individualized specific antigen peptides for each patient through the multi-target prediction of antigens, which may offer precision potential in treating different cancer patients.

Multiple peptide-pulsed DCs combined with CTLs therapy provides a precise targeted therapy for solid tumors by infusing immune cells (18). Herein, the peripheral blood from patients was used to analyze the specific peptides of the tumor by comparing the pool of peptides. Multiple peptides were then pulsed into DCs to induce antigen-specific CTLs. To explore the potential anti-tumor function of DC-CTLs, the secretion of IFN-γ was detected by enzyme-linked immune absorbent spot (ELISPOT) assay. Furthermore, five patients were enrolled in this study to evaluate the efficacy and safety of multiple peptide-pulsed DCs combined with CTLs therapy. The production of IFN-γ and other intracellular cytokines including IL-4, IL-2, perforin, and Granzyme B were also analyzed in PBMCs derived from patients before and after one month cell infusion. Our study may provide a novel optional choice for patients with advanced solid tumors.

## Materials and methods

### Patients

Five cancer patients were recruited at the Biotherapy Center of the First Affiliated Hospital of Zhengzhou University from November 2020 to June 2021. All patients had signed an informed consent form before participating in the study. This study was performed in accordance with the ethical guidelines of the Declaration of Helsinki and approved by the Ethics Committee of the First Affiliated Hospital of Zhengzhou University (2020-KY-364). The recruitment criteria were as follows: 1. patients were pathologically diagnosed with cancer without abnormalities in liver and kidney function; 2. the Karnofsky performance status was > 70 years, and the age ranged from 18 to 75 years; 3. None of patients had autoimmune diseases, virus infections (HIV, HCV, or HBV), or blood diseases. Sex, age, tumor type, and other clinical parameters of the enrolled patients are summarized in **Table 1**.

**Table 1:** Patient demographics and outcomes.

Patient no.	Age	Sex	Tumor	lymphatic metastasis	Tumor stage	previous treatment	Doses DCs	Doses CTLs	DFS/PFS (months)	OS (months)	Patient status
1	70	F	colorectal cancer	N	IIA	Surgery +Chemotherapy	8.6×10^6^	1×10^10^	28.2	32.5	Alive
2	66	M	melanoma	Y	IV	Surgery+Anti PD-1 antibody	1.5×10^7^	3.4×10^9^	*	31.1	Alive
3	60	F	breast cancer	Y	IV	Surgery +Chemotherapy	3.8×10^6^	1×10^10^	*	1.7	Dead
4	47	F	lung cancer	N	IA	Surgery	3.3×10^6^	2.9×10^9^	20.2	28.7	Alive
5	24	F	colorectal cancer	Y	IV	Surgery +Chemotherapy	6×10^6^	2.8×10^9^	17.6	26.3	Alive

M, male; F, female; N, no; Y, yes; DFS, Disease Free Survival; PFS, Progression Free Survival; OS, Overall Survival; *, no efficacy evaluation.

### Screening and preparation of antigenic peptides in patients

Five milliliters of peripheral blood were collected from patients without the use of anticoagulants. The blood samples were processed to separate the serum. Acetonitrile solution and hydrophile-lipophile balance (HLB) cartridges were utilized to filter and remove lipids, high-abundant proteins, and low-molecular-weight metabolites, resulting in the isolation of the target polypeptide. The extracted serum peptidome samples were then subjected to Q-TOF MS (Waters Acquity Ultra-Performance Liquid Chromatography/Xevo G2 QTOF) analysis. The acquired subsequent peptide targets were analyzed using a bio-high-technology DC-CTLs tumor-specific targets tumor database. Subsequently, individual antigens for each patient were obtained, and all the identified multiple peptides against antigens (**Table 2**) were synthesized for the culture of DC-CTLs.

**Table 2:** The type of peptides for each patient.

Patient No.	Name of Peptides	Number of peptides
1	BHCr1010, BHCr1809, BHCr2208, BHCr0410, BHCr2910, BHCr2410, BHCr0310	7
2	BHme1710, BHme0410, BHme1110, BHme1410	4
3	BHBr1710, BHBr1008, BHBr1610, BHBr0710, BHBr1110, BHBr1510	6
4	BHNS2710, BHNS0810, BHNS1210, BHNS1710, BHNS1810, BHNS2309, BHNS1510	7
5	BHCr1510, BHCr3008, BHCr0410, BHCr1809, BHCr1909	5

### Generation of mature DCs

For each patient with cancer, about 8×10^7^-1×10^8^ peripheral blood mononuclear cells (PBMCs) were obtained from 50-60 mL blood by using Ficoll density gradient (Tianjin, HY, China). Cells were washed twice with saline, resuspended at a density of 2 × 10^6^ cells/mL in GT-551 serum-free medium (Takara, Japan), and cultured for 2 h at 37 °C with 5% CO_2_. The adherent cells were induced to DCs by adding 500 U/mL recombinant human interleukin-4 (rhIL-4, CellGenix, Germany), 1000 U/mL recombinant human granulocyte-macrophage colony (rhGM-CSF, Liaoning WX, China) and 100 ng/mL tumor necrosis factor-α (TNF-α, R&D Systems, USA) to the GT551 medium containing with 50 μg/mL tumor antigen peptides. On Day 5, all mature DCs were harvested and half of them were taken out and infused into patients.

### Generation of DC-CTLs

To stimulate the expansion of specific CTLs, the remaining non-adherent cells from PBMCs of each patient were added the 1000 U/mL IFN-γ (Beijing SL, China), 100 ng/mL anti-CD3 antibody (Boehringer Mannheim, Germany) and 1000 U/mL recombinant human IL-2 (rhIL-2, Beijing SL, China) within GT551 medium. On Day 5, these suspended cells were co-cultured with half of mature DCs. Fresh autologous serum culture medium containing IL-2 was added every 2-3 days.

### Phenotypic analysis of cultured cells

To evaluate the maturation of DCs during culture, the single-cell suspensions containing DCs before and after culture were washed with phosphate buffered saline (PBS) containing 2% fetal bovine serum (FBS) and were then stained with CD14 (PerCP, clone no. HCD14), CD11c (Brilliant Violet 421™, clone no. Bu15), CD80 (FITC, clone no. 2D10), CD86 (PE/Cyanine7, clone no. IT2.2), CD83 (PE, clone no. HB15e), and HLA-DR (APC/Cyanine7, clone no. L243) antibodies under dark conditions. After incubation at 4 °C for 15 min, the phenotype of DCs were detected by flow cytometry. The specific CTLs were collected on day 14 and stained with antibodies against CD3 (PE/Cyanine7, clone no. OKT3), CD4 (PerCP, clone no. RPA-T4), CD8 (APC/Cyanine7, clone no. SK1), CD56 (PE, clone no. 5.1H11), PD-1 (FITC, clone no. EH12.2H7), CTLA-4 (APC, clone no. BNI3), TIM-3 (Brilliant Violet 421™, clone no. B8.2C12), CD69 (PE, clone no. FN50), and CD28 (Brilliant Violet 650™, clone no. CD28.2 USA). All antibodies were purchased from BioLegend (San Diego, USA). Levels of these markers were measured using a FACSCanto II flow cytometer (BD Biosciences).

### ELISPOT assay

An IFN-γ ELISPOT kit (Dakewe, China) was used to evaluate the immunogenicity of DC-CTLs or PBMCs according to the manufacturer’s instructions. Briefly, PBMCs or DC-CTLs (2×10^5^/well) were stimulated with per antigen peptide (50 μg/mL) by using 96-well culture plate pre-coated with IFN-γ antibody. Meanwhile, the phytohemagglutinin (PHA) group served as a positive control, and cells without antigenic peptides served as a negative control. The 96-well plate was incubated at 37 °C with 5% CO_2_ for 16 h. Cells were removed and the biotinylated antibody was added to each well (37 °C for 1 h), followed by adding the enzyme-linked avidin (37 °C for 1 h), and then the AEC solution mix was added to the plate at room temperature (30 min). Finally, deionized water was added to stop the color rendering. The spots were quantified using a Mabtech IRIS FluoroSpot/ELISPOT reader.

### Phenotype of lymphocytes in the peripheral blood

Peripheral blood was obtained from the patients before and after cells infusion. Antibodies against CD3 (PE/Cyanine7, clone no. OKT3), CD4 (PerCP, clone no. RPA-T4), CD8 (APC/Cyanine7, clone no. SK1) and CD56 (PE, clone no. 5.1H11) were used to stain the PBMCs, which were then incubated with FACS lysis buffer for 15 min. The supernatant was removed and the cells were resuspended in FACS lysis buffer. Finally, lymphocyte phenotypes were detected using flow cytometry.

### Cytokine production analysis

To analyze the production of cytokines, the PBMCs from patients were stimulated with phorbol 12-myristate 13-acetate (PMA, 1 μg/mL; Sigma-Aldrich), ionomycin (1 μg/mL; Sigma-Aldrich) and brefeldin A (5 μg/mL; BioLegend, USA) for 6 h. The cells were washed with PBS and then stained with antibodies (BioLegend, USA) including PE/Cyanine7 anti-human CD3 (OKT3), PerCP anti-human CD4 (RPA-T4), APC/Cyanine7 anti-human CD8 (SK1), and PE anti-human CD56 (5.1H11) at 4 °C for 15 min followed by 4% formalin, subsequently. Then the cells were incubated with permeation buffer at 4 °C for 30 min, and the IL-4 (FITC, clone no. MP4-25D2), IL-2 (PE, clone no. MQ1-17H12), IFN-γ ( APC, clone no. 4S.B3), perforin (APC, clone no. dG9), and Granzyme B (FITC, clone no. QA16A02) antibodies were used for intracellular cytokine staining.

### T cell receptors sequence analysis

TCR libraries were constructed and next-generation sequencing (NSG) was performed by Chengdu ExAb Biotechnology Co, Ltd as previous report (19). Briefly, the genomic DNA was extracted using the QIAGEN OneStep reverse transcription polymerase chain reaction (RT-PCR) Kit (QIAGEN), following the manufacturer’s instructions. The PCR products were purified by using Agencourt AMPure XP beads (Beckman). Extracted genomic DNA was accurately quantified using a Qubit 1X dsDNA HS Assay Kit (Invitrogen). The TCRβ complementarity-determining region 3 (CDR3) regions were amplified by Multiplex PCR and sequenced on an Ion GeneStudio S5 System (Thermo Fisher Scientific). The TCRβ CDR3 regions were discerned based on the definition established by the International ImMunoGeneTics (IMGT) Collaboration, and the V, D, J segments contributing to each CDR3 region were identified by a standard algorithm. For each sample, we used the Shannon-Weaver index (20) and the diversity 50 (D_50_) value to estimate the TCR diversity. The Shannon-Weaver index reflected the diversity of the TCRs. The D_50_ index was used as a measure of the diversity and clonality of the TCRβ repertoire and defined as the proportion of TCRβ CDR3 clonetypes that account for the cumulative 50% of the total TCRβ sequences in the sample (21).

### Treatment plan

Each patient for cell therapy, 50-60 mL of fresh peripheral blood was collected from patient. Mature DCs were harvested, and half of them were infused into patients on day 5, with a total number of 3.3×10^6^ to 1.5×10^7^ cells. The remaining mature DCs were co-cultured with lymphocytes and collected on the 14^th^ day and 15^th^ days. The DC-CTLs were injected intravenously within 30 min at a total number of 2.8×10^9^ to 1×10^10^ cells. All the infused cells were washed and suspended in 100 mL saline containing 20% human serum albumin and were confirmed to be free of bacterial, mycoplasmal or fungal contamination, and endotoxins were less than 0.25 EU/mL.

### Assessment of adverse effects, and clinical efficacy

Safety evaluations were performed by assessing the incidence of clinical adverse events, which were graded using the National Cancer Institute Standard for Common Terminology Criteria for Adverse Events, Version 5.0. Patients completed a simplified questionnaire on quality of life after cells infusion that recorded changes in appetite, sleep, weight, memory, depression, energy, anxiety, and mobility. A change for the better was considered an improvement in quality of life. As of August 1, 2023, we have followed up the patients for more than 32 months and evaluated their clinical responses. Disease free survival (DFS) was defined as the time without disease recurrence or progression after the cell infusion. Progression free survival (PFS) was defined as the time between the onset of cell transfusion and disease progression or death from any cause.

### Statistical analysis

Data were plotted using GraphPad Prism version 8.0 and Photoshop version CS5. The results are expressed as the Mean ± standard error of the mean (SEM). Student’s t-test was used to test the statistical significance of the mean difference between groups, and *P* < 0.05 was considered statistically significant.

## Results

### Patient characteristics

**Figure 1:**
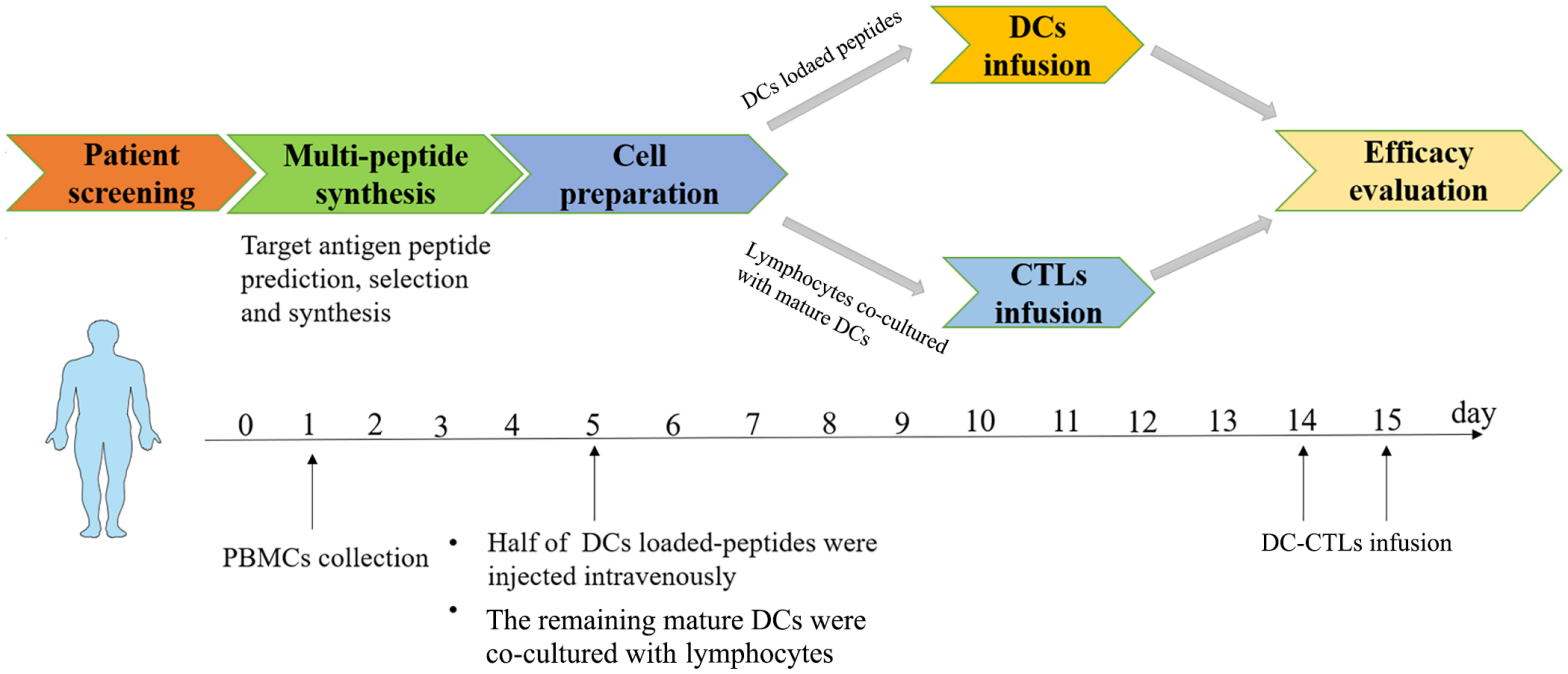
Basic flow chart of multiple peptides-pulsed DCs combined with CTLs therapy. Autologous cells were obtained from each patient. DCs plus multiple peptides were cultured for 5 days and then co-cultured with CTLs for 10 days. DCs were infused on the 5^th^ day, DC-CTLs were then respectively transfused to their respective patients on the 14^th^ and 15^th^ days, and the efficacy of specific DC-CTLs was observed both *in vitro and in vivo*. Side effects and follow up were also observed for each patient.

From November 2020 to June 2021, five cancer patients (1 male and 4 females) with a median age of 57 years (range: 24-70 years) who had undergone traditional treatments were recruited for this study (**Table 1**). The treatment details and entire management process of multiple peptides-pulsed DCs combined with CTLs therapy, including patients screening, multi-peptide synthesis, blood collection, and cells infusion, are shown in **Figure 1**. Patients 1, 3 and 4 did not receive any other anti-tumor treatments after receiving peptide-pulsed DCs combined with CTLs therapy. Patient 2 was treated with anti PD-1 antibody for two years. Patient 5 was underwent ablation of the lung and liver metastases.

### Phenotypic analysis of DCs and DC-CTLs

**Figure 2:**
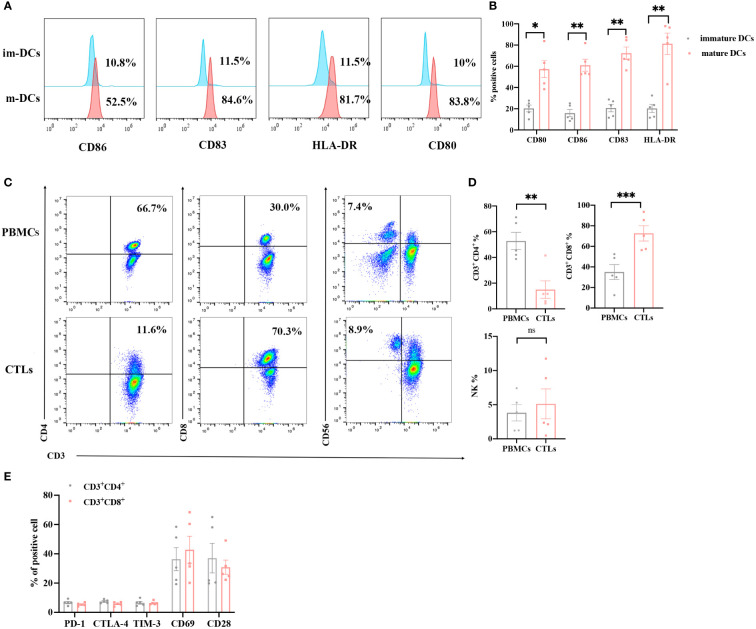
Phenotypes of DCs and DC-CTLs. **(A, B)** The expression of CD80, CD86, CD83 and HLA-DR were detected in immature DCs on day 0 and mature DCs on day 5 by flow cytometry. **(C, D)** The population of DC-CTLs and PBMCs, rates of CD3^+^CD4^+^, CD3^+^CD8^+^, and CD3^-^CD56^+^ cells in DC-CTLs and PBMCs. **(E)** The expression of CD28, CD69, PD-1, CTLA-4 and TIM-3 in DC-CTLs were tested by flow cytometry. (*P < 0.05, **P < 0.01, ***P< 0.001, Student’s t test).

Mature DCs can effectively present antigens to DC-CTLs. We obtained DCs from the peripheral blood of patients, and cultured these to maturation *in vitro*. On the day 5 of cultivation, we analyzed the phenotypes of the DCs using flow cytometry. The expression of CD80, CD86, CD83 and HLA-DR in the cultured DCs were significantly upregulated compared with that in the pre-culture, suggesting that DCs acquired a mature phenotype (**Figures 2A, B**). The half of mature DCs were then co-cultured with CTLs on day 5 to confer DC-CTLs antigen specificity. After 14 days culture, the proportion of immune cell subsets and the level of activated/inhibited markers were detected in DC-CTLs. Compared with PBMCs, the proportion of CD3^+^CD4^+^ cell subset in DC-CTLs decreased. The CD3^+^CD8^+^ cell subset was upregulated, and CD3^-^CD56^+^ (NK) cell subset was increased slightly (**Figures 2C, D**). We also found that the DC-CTLs expressed a high levels of activation markers such as CD69 and CD28, but had low levels of inhibition markers, including PD-1, TIM-3 and CTLA-4 (**Figure 2E**).

### DC-CTLs function detection by ELISPOT

**Figure 3:**
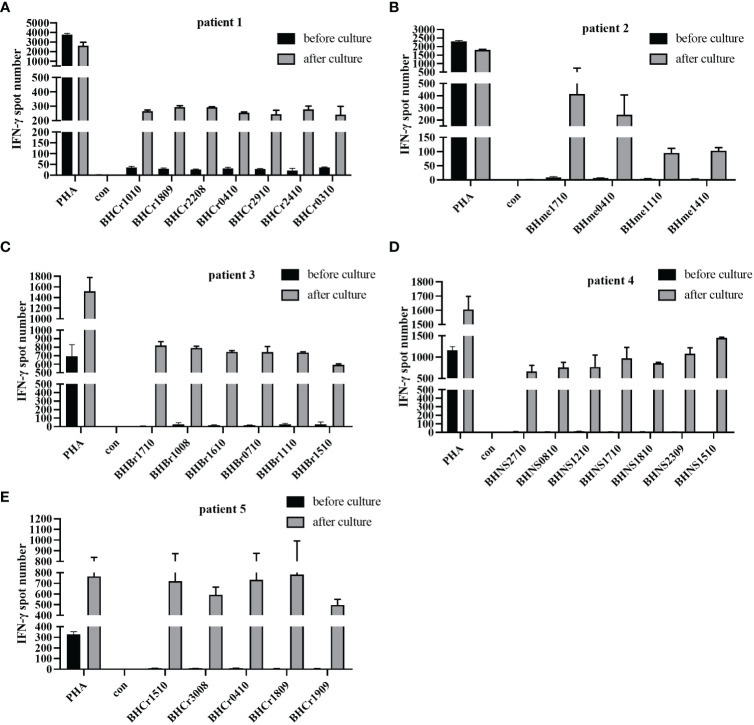
IFN-γ ELISPOT assay was used to detect the anti-tumor function of PBMCs and DC-CTLs against specific antigens. **(A–E)** Autologous PBMCs and DC-CTLs were stimulated with multiple peptides for 16 h, PHA and peptide-free stimulation represented the positive control and negative control, respectively.

Several studies have reported that the IFN-γ ELISPOT assay was performed to assess the T cell specific antigen response (14, 22). To further verify the identified peptides have the ability to induce tumor specific CTL activity, the PBMCs of patients were used to detect the IFN-γ secretion, as well as the DC-CTLs. Among the 5 patients, the number of IFN-γ spots in cultured CTLs was much higher than that in uncultured cells (**Figures 3A–E**), which exhibited that the identified peptides have the ability to induce activated tumor specific CTLs.

### Evaluation of patient’s immune status

The immune status of patients is closely related to the disease progression and the immune function of patients may be reinforced in different ways after cell transfusion (23). To evaluate changes in immune status, flow cytometry was used to detect cell subsets and endogenous factors in PBMCs from patients before cell therapy and one month after cell transfusion. After treatment, the proportion of CD4^+^/CD8^+^ was slightly elevated, and the ratio of CD3^-^CD56^+^ (NK) cells was also upregulated. These results display that multiple peptides-pulsed DCs combined with CTLs treatment could strengthen the immune status of cancer patients, at least for a short period of time (**Table 3**).

**Table 3:** Peripheral lymphocyte subset assay before and after treatment.

Group	Cell treatment		
	Before treatment	After treatment	*P* value
CD3^+^	69.32 ±6.20	76.53 ± 2.54	0.241
CD3^+^ CD4^+^	48.19 ± 6.31	52.88 ± 5.13	0.035
CD3^+^ CD8^+^	40.70 ± 5.77	36.94 ± 4.33	0.186
CD4^+^ / CD8^+^	1.31 ± 0.34	1.55 ± 0.35	0.020
CD3^-^ CD56^+^	4.47± 1.30	5.89 ± 2.11	0.418

**Figure 4:**
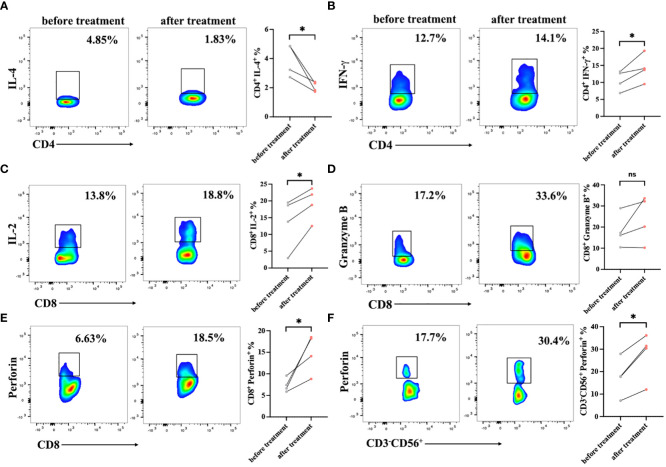
Cytokine production in the peripheral blood lymphocytes of patients before and after cell therapy. PBMCs were stimulated with PMA, ionomycin and BFA for 6 h, and were then stained with IL-2, IFN-γ, Perforin, and Granzyme B fluorescence-labeled antibodies. **(A, B)** Frequencies of IL-4 and IFN-γ in CD3^+^CD4^+^ subsets of PBMCs. **(C–E)** Frequencies of IL-2, Perforin, Granzyme B in CD3^+^CD8^+^ subsets of PBMCs. **(F)** Frequencies of Perforin in CD3^-^CD56^+^ subsets of PBMCs. The results are represented as Mean ± SEM values. (*P < 0.05, ns, no significance, Student’s t-test).

To verify the immune cell function of patients, the expression levels of cytokines were detected in PBMCs by intracellular staining using a flow cytometer. The serection of intracellular factors including IL-4, IFN-γ, perforin and Granzyme B in PBMCs of patients before and after treatment were detected. After treatment, the CD3^+^CD4^+^ T cell function was improved with lower IL-4 expression and higher IFN-γ secretion (**Figures 4A, B**). Meanwhile, we observed a high production of IL-2 and perforin in CD3^+^CD8^+^ cells after cells infusion, but there was no significance of Granzyme B expression in CD3^+^CD8^+^ T cells before and after DC-CTLs treatment (**Figures 4C–E**). These results indicated that CD3^+^CD8^+^ cells had a more potent cytotoxic function after treatment. Furthermore, CD3^-^CD56^+^ cells were activated with higher expression of perforin (**Figure 4F**). This showed that multiple peptides-pulsed DCs combined with CTLs therapy play a crucial role in actively regulating the immune system of patients with cancer.

### Specific immune responses

**Figure 5:**
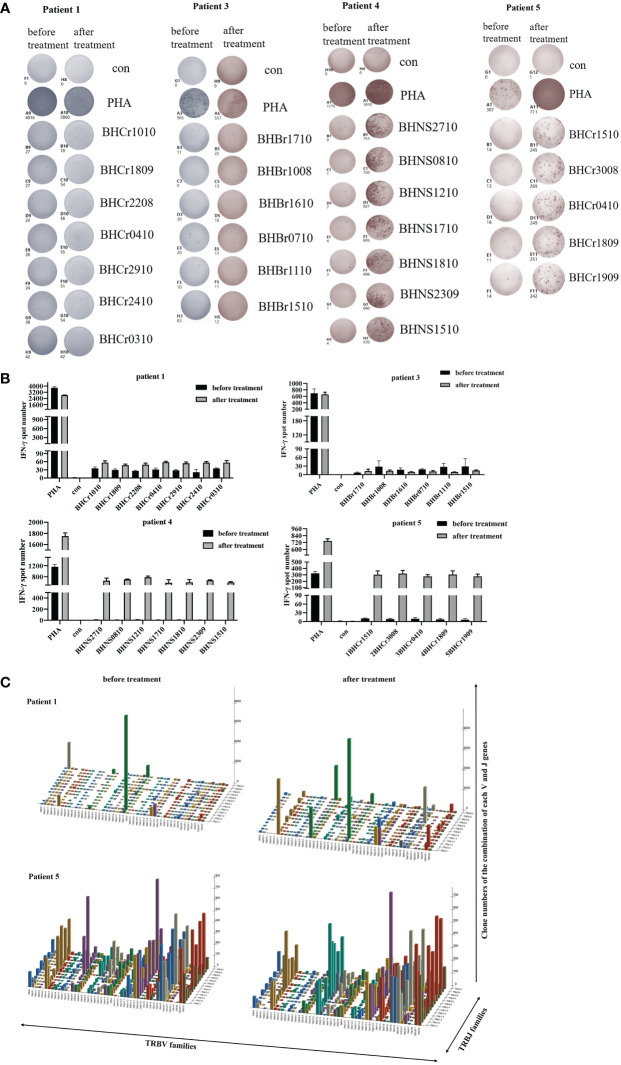
Immune responses of patients to personalized peptides. **(A)** The IFN-γ ELISPOT images of the patient’s PBMCs before and after treatment. **(B)** Statistics showed the number of spots for each individual antigenic peptide. **(C)** Three-dimensional images of TCRβ chain repertoires: results from TCRs repertoire identifying the CDR3 amino acid sequences and expression levels of T-cell receptor beta variable gene segment (TRBV) and T-cell receptor beta joining gene segment (TRBJ) region genes. The x and y axes showed the combination of V and J genes (TRBV and TRBJ families), and the z axis showed their clone numbers of usage.

To evaluate the immune responses against the antigen peptides, PBMCs were collected and analyzed using an ELISPOT assay after one month of cells infusion. The number of IFN-γ spots produced in the peripheral blood of each patient for the antigen peptides was shown in **Figures 5A, B**. The presence of antigen-specific T cells in each patient varied with respect to the presence and magnitude of the response. The PBMCs of patients 1, 4, and 5 became more responsive to antigen peptides after treatment, whereas patient 3 continued to have progressive disease and showed no antigen-specific reactions. Patient 2 declined to provide blood samples after cells infusion. Our results suggest that DC-CTLs therapy may not be beneficial for patients with end‐stage disease. However, the earlier the DC-CTLs therapy is received, the better response to the treatment is to be expected.

### Clinical response

The follow-up results showed that four patients were alive, and one patient failed to halt the progression of disease. The overall survival (OS) time of patients ranged from 1.7 month to 32.5 months (**Table 1**). Patient 1 with colorectal cancer had a DFS of 28.2 months, and she experienced a dramatic drop in serum carbohydrate antigen 724 (CA724) levels after DC-CTLs therapy from 24.6 U/mL to 15.46 U/mL. The number of circulating tumor cells (CTCs) in 5mL peripheral blood decreased from 4 to 1 a month after infusion. Patient 2 with melanoma who had lymph node metastasis before cell therapy remained alive after 31.1 months of follow-up. But he declined to undergo CT or MRI to evaluate the tumor. Patient 3 subsequently undergone DC-CTLs therapy after failure of previous surgery and chemotherapy. She was a breast cancer patient with multiple metastases and high levels of carcinoembryonic antigen, who was originally in the stage of rapid disease progression prior to enrollment. The patient ultimately succumbed to disease progression and dead 1.7 month after cells infusion. Patient 4 with lung cancer had a DFS for 20.2 months after DC-CTLs therapy. The number of CTCs in 5 mL peripheral blood in patient 4 fell off sharply from 16 to 1 three months after cells infusion. Patient 5, with colon cancer who had lung metastases before enrolment, underwent DC-CTLs therapy because of intolerable alimentary tract adverse reactions caused by chemotherapy. She had a progression free survival of 17.6 months after cells infusion (**Table 1**). Our results suggest that patients had a long survival time after DC-CTLs therapy, indicating multiple peptides-pulsed DCs combined with CTLs therapy may provide an individual treatment strategy for patients with cancer.

### T cell receptor repertoire analysis

TCR diversity and the combination of the V, D and J genes reflect T cell function and immune status. Analysis of CDR3, which is based on V-D-J gene rearrangement and is responsible for identifying antigens, has been used to evaluate T cell clonality and diversity (19, 24). Structural diversity of the TCR repertoire was directly observed by deep sequencing of the TCRβ CDR3 region. Therefore, we used quantitative TCRβ sequencing to examine the changes in T cell diversity and clonality before and after treatment in patients. Our results found that the Shannon-Weaver and D_50_ index of the patients 1 and 5 were slightly increased (**Table 4**) after DC-CTLs therapy, as well as the number of TCR clones (**Figure 5C**). The other patients were not tested in this study. Thus, we infer that the increased TCRβ diversity is potentially associated with the clinical response of DC-CTLs therapy for patients with cancer.

**Table 4:** Variation of the D_50_ and Shannon-Weaver index before and after treatment.

Patient no.	Before treatment	After treatment	Before treatment	After treatment
1	0.000938	0.001859	5.48	6.17
5	0.196146	0.206543	9.11	9.14

### Quality of life and adverse effects

To measure subjective responses to treatment, we analyzed several parameters related to quality of life and existing suffering after cells infusion (**Table 5**). Improvements of life quality were reported some patients, including advanced cases, such as better appetite (three patients; 60%), improved sleep (three patients; 60%), weight gain (three patients; 60%), and improved memory (three patients; 60%). These results showed that patients’ subjective sensations and quality of life were improved after DC-CTLs treatment.

**Table 5:** Improved quality of life after DC-CTLs infusion.

	Change in general status, number of patients(%)		
	Better	No change	Worse
Appetite	3(60)	1(20)	1(20)
Sleeping	3(60)	1(20)	1(20)
Body weight	3(60)	2(40)	0(0)
Memory	3(60)	2(40)	0(0)
Depression	4(80)	1(20)	0(0)
Energy	3(60)	2(40)	0(0)
Anxiety	4(80)	1(20)	0(0)
Mobility	3(60)	2(40)	0(0)

Adverse effects, including fever, fatigue, muscle aches, chills, rash, allergies, laryngeal edema, phlebitis, and lysis syndrome, were analyzed. Treatment-related grade 1 or grade 2 adverse events were observed in 1/5 (20%) of patients. The most common side effects were mild, and spontaneous remission occurred several hours after cells infusion. No grade 3 or 4 adverse effects were observed (**Table 6**). None of the patients discontinued therapy because of intolerable side effects.

**Table 6:** Side effects after DC-CTLs infusion.

Event	Grade 1 or 2 Number of patients (%)	Grade 3 or 4
Fever	1(20)	0
Fatigue or sore	1(20)	0
Muscular soreness	0	0
Chill	0	0
Rash	0	0
Allergy	0	0
Laryngeal edema	0	0
Phlebophlogosis	0	0
Lysis syndrome	0	0

## Discussion

Numerous clinical trials have indicated that DC-CTLs therapy can be used for the treatment of hepatocellular carcinoma, kidney cancer, gastric cancer and other solid tumors (13, 14, 25–27). However, the DC-based T cell therapy has some limitations: 1. how to screen and identify individual peptides; 2. whether DCs loaded with antigen peptides can effectively present tumor antigens to T cells (28, 29). In our study, a novel technology was used to identify tumor specific target peptide pools, whose molecular structures were distinctive from those known tumor antigens (see patent disclosure of CN 104655849 B) (30). The individual tumor antigen peptides of each patient with cancer were developed by differential protein screening for identifying dominant MHC binding peptide epitopes from the database of Hebei Bio-technology Co., Ltd (30). Furthermore, the efficacy and safety of peptides-pulsed DCs combined with specific T cells were evaluated both *in vitro* and *in vivo*.

Mature DCs can effectively stimulate the proliferation of naive T cells and induce T cells to become the antigen specific CTLs (31, 32). In our study, DCs were stimulated with multiple peptides, and the mature markers such as CD83, CD80, CD86 and HLA-DR were significantly upregulated on Day 5, suggesting that the DCs acquired a mature phenotype and may induce the production of specific CTLs. CTLs can directly kill tumor cells quickly, efficiently and specifically through the cytokine secretion (33, 34). As reported, the secretion of IFN-γ was associated with the response of tumor-specific T cells, and exerts anti-tumor effects by inhibiting tumor-cell proliferation and promoting tumor cell apoptosis (35–38). As a consequence, the production of IFN-γ exhibits an intensely synergistic boost to antigen-specific immune responses during immunotherapy (39). Our results showed that specific-CTLs, after co-culture with multiple peptides-pulsed DCs, can secret high levels of IFN-γ than that of PBMCs from patients before treatment, demonstrating that DC-CTLs have been activated and may produce a clinical benefit for patients by targeting antigenic peptides after culture. Meanwhile, the DC-CTLs expressed high levels of activation markers, such as CD69 and CD28, while having low levels of molecules including PD-1, TIM-3 and CTLA-4. The expression of these inhibitory markers on T cells, which contributes to a dysfunctional T-cell response with an “exhausted” phenotype (40–42). Our results indicated that antigen-specific T cells were massively expanded and activated.

The CD4^+^/CD8^+^ ratio is an indicator of chronic infection and immune activation. A decreased or inverted CD4^+^/CD8^+^ ratio represents poor immune status, which could be used as a predictor of severe infection or malignancy (43–45). We found that the ratio of CD4^+^/CD8^+^ slightly increased in the PBMCs of patients after treatment. Several studies have also demonstrated that the secretion of pro-inflammatory cytokines represents the effector function of T cells (46, 47). Herein, we found that the secretion of IL-2, IFN-γ and perforin was increased, while the proportion of IL-4 was decreased in PBMCs of patients who received cell therapy. These results showed that the immune status of patients with cancer was improved, which may be associated with the anti-tumor function of DC-CTLs. Meanwhile, our results exhibited that the level of IFN-γ was significantly upregulated in the patient’s lymphocytes upon stimulation with DC loaded peptides after treatment, indicating that specific DC-CTLs were activated and acquired an anti-tumor function. Notably, a diverse repertoire of T cells directed against tumor-specific targets is a key factor impacting the success of immunotherapy (48, 49). Furthermore, TCR repertoire during immunotherapy will be highly valuable for understanding anti-tumor response and ultimately for the development of new strategies to improve outcomes of patients with cancer (50). Herein, we found that the diversity of patient’s TCRβ chain increased after DC-CTLs therapy, which may serve as evidence of a better response to cell therapy.

In addition, among all the patients treated with multiple peptides-pulsed DCs combined with CTLs, one of the patients had a DFS period of up to 28.2 months. No severe toxicities were observed, and only one patient experienced disease progression in a short time, suggesting that the treatment was safe and effective. However, immunotherapy differs from chemotherapy, which may require a relatively long term to evaluate its clinical benefits. The limited number of patients prevents us from drawing definitive conclusions. To further evaluate the impact of multiple peptides-pulsed DCs combined with CTLs therapy on patients with cancer, we need to recruit more patients to continue the clinical trials.

In conclusion, our cultured mature DCs carried personalized tumor antigen-derived peptides, and CTLs primed with these DCs exhibited high cytotoxicity against tumor targets. We evaluated the effects of multiple peptide-pulsed DCs in combination with CTLs therapy. Most patients were able to prevent progression of cancer, and there is no grade 3 or 4 adverse events occurred during the follow up period after cell transfusion. The quality of life in patients was either preserved or improved. Our results showed that the combination of multiple peptides-pulsed DCs and CTLs therapy could prolong survival and improve the quality of life, which has the potential to act as an individualized treatment strategy for cancer.

## Data availability statement

The original contributions presented in the study are included in the article/supplementary material. Further inquiries can be directed to the corresponding authors.

## Ethics statement

The studies involving humans were approved by the Ethics Committee of the First Affiliated Hospital of Zhengzhou University. The studies were conducted in accordance with the local legislation and institutional requirements. The participants provided their written informed consent to participate in this study.

## Author contributions

XZ: Conceptualization, Funding acquisition, Writing – original draft. ZZ: Writing – original draft, Data curation. CW: Data curation, Writing – original draft, Formal Analysis. JH: Data curation, Formal Analysis, Writing – review & editing. SY: Formal Analysis, Writing – review & editing. JL: Formal Analysis, Writing – review & editing. HG: Writing – review & editing, Methodology. BP: Methodology, Writing – review & editing. ZL: Methodology, Writing – review & editing. YZ: Writing – review & editing, Conceptualization, Funding acquisition.
